# Translating mental health diagnostic and symptom terminology to train health workers and engage patients in cross-cultural, non-English speaking populations

**DOI:** 10.1186/s13033-017-0170-2

**Published:** 2017-10-03

**Authors:** Bibhav Acharya, Madhur Basnet, Pragya Rimal, David Citrin, Soniya Hirachan, Sikhar Swar, Poshan Thapa, Jagadamba Pandit, Rajeev Pokharel, Brandon Kohrt

**Affiliations:** 1 Possible, Bayalpata Hospital, Sanfebagar-10, Achham, Nepal; 2 Department of Psychiatry, University of California, San Francisco, San Francisco, CA USA; 3 Shared Minds, Boston, MA USA; 4 Faculty of Medicine, Department of Psychiatry, B.P. Koirala Institute of Health Sciences, Dharan, Sunsari Nepal; 5 Department of Anthropology, University of Washington, Seattle, WA USA; 6 Department of Global Health, University of Washington, Seattle, WA USA; 7 Henry M. Jackson School of International Studies, University of Washington, Seattle, WA USA; 8 Department of Psychiatry, University of Minnesota, Minneapolis, MN USA; 9 Psychiatric Department, Kathmandu Medical College, Kathmandu, Nepal; 10 Ministry of Health, Policy Planning & International Cooperation Division, Kathmandu, Nepal; 11 Transcultural Psychosocial Organization Nepal, Kathmandu, Nepal; 12 Department of Psychiatry, George Washington University, Washington D.C., USA

**Keywords:** Mental health, Global health, Nepal, Partnerships, Low- and middle-income countries

## Abstract

Although there are guidelines for transcultural adaptation and validation of psychometric tools, similar resources do not exist for translation of diagnostic and symptom terminology used by health professionals to communicate with one another, their patients, and the public. The issue of translation is particularly salient when working with underserved, non-English speaking populations in high-income countries and low- and middle-income countries. As clinicians, researchers, and educators working in cross-cultural settings, we present four recommendations to avoid common pitfalls in these settings. We demonstrate the need for: (1) harmonization of terminology among clinicians, educators of health professionals, and health policymakers; (2) distinction in terminology used among health professionals and that used for communication with patients, families, and the lay public; (3) linkage of symptom assessment with functional assessment; and (4) establishment of a culture of evaluating communication and terminology for continued improvement.

## Background

When working in cross-cultural settings, mental health professionals (clinicians, researchers, and educators) will encounter the need for clinical translation from English to other languages. The issue of translation is particularly salient when working with underserved populations in three common settings: English-speaking professionals engaging with health workers and patients in low- and middle-income countries (LMICs); high-income country (HIC)-based English-speaking professionals interfacing with people and patients in HIC who retain strong cultural and linguistic ties from LMICs (e.g., recent immigrants, travelers, and refugees); and LMIC-based professionals receiving undergraduate and post-graduate medical education in English (e.g., medical students and residents using English textbooks and protocols) but interacting with patients and colleagues in their home countries in their native language.

In each of these settings, appropriate translation is a critical step in ensuring fidelity and cross-cultural adaptation of the training, clinical, and other communication materials. There is often a lack of equivalent terms like “depression” or “psychosis” in local languages. Other illnesses, like hypertension or diabetes, also frequently lack corresponding terms in other languages. However, terms used for mental illnesses and their symptoms are highly affected by socio-cultural and linguistic parameters, elevating the importance of approaching equivalence. In addition, given that mental health diagnoses are made via assessment of speech, behaviors, and emotions (rather than with a lab result or an imaging study), it is critical that translated terms for both the diagnoses and symptoms are aligned with the health workers’ and patients’ understanding and conceptualization.

Although professional medical interpreters may be available in some settings (e.g., when HIC-based clinicians cater to non-English speaking patients, or HIC-based researchers work in LMICs), they will still need training in appropriate translation and adaption of mental health protocols and educational materials. Guidelines for rigorously cross-culturally adapting and validating clinical tools and psychiatric scales are available and have been widely used [1, 2]. These methods include translating, back-translating, resolving disagreements, piloting tools with populations with and without the disorder, and conducting blinded quality-control with a structured diagnostic interview. These steps also include qualitative inquiry with patients, families, and community members to generate a specific understanding of the cultural conceptualization of mental illness and well-being in the target population. Examples of the methods for completing such formative work on concepts of mental disorders using individual interviews, focus group discussions, vignettes, and ethnography are described elsewhere [3, 4].

However, there are not similar standardized approaches for determining the language that health professionals use to communicate with one another (e.g., when an LMIC-based health worker receives mental health training) nor guidelines recommending how clinicians should communicate with patients and the public who are non-English speakers. Whereas researchers dedicate considerable energy to assuring the generalizability of psychiatric questionnaire results, the terminology used in training mental health specialists and non-specialist health workers and the subsequent communication they provide to patients can vary widely among clinicians and institutions. Below, we share our challenges in mental health translation for terminology used by health professionals and in health settings, and we make four recommendations for harmonization of terminology, distinguishing professional communication from patient communication, linking terminology with functional assessments, and establishing a culture of evaluating communication for quality improvement.

## Common challenges and recommendations

Based on our experience as clinicians, researchers, and educators based in LMICs and HICs, we present challenges in translation of clinical materials in mental health, and provide case examples and recommendations for each.

### Harmonize diagnostic and symptom terminology among clinicians, educators of health professionals, and health policymakers

Mental health professionals, other healthcare workers, and policymakers working independently may develop various translations of the same concepts, diagnoses, and symptoms. Uncoordinated work and a lack of a commonly used bilingual lexicon can create challenges for health workers. Although standardized research methods for producing transcultural translations have been widely used in Nepal [2], these are less frequently employed by clinicians and in health worker trainings. For example, although extensive qualitative and quantitative rigor was employed for cultural translation and clinical validation of the PHQ-9 [2], the wording established may not be consistently used by non-specialist and specialist health workers who may have idiosyncratic approaches to diagnostic interviews, psychoeducation, and training [5]. This is not dissimilar from the diversity of approaches that clinicians may take when discussing a condition in English.

As an example, the term “schizophrenia” or the generic term “psychosis” is translated in various ways in Nepal. A lay person may understand a commonly used term “*paagalpan*,” which translates to “the state of being mad” but evokes a highly stigmatizing term that carries the pejorative quality of the word “crazy”. Given the challenge of capturing the presentation of psychosis in a non-stigmatizing manner, many Nepali psychiatrists simply do not provide a diagnosis to the patient or may use the term “schizophrenia” without seeking a Nepali equivalent. However, for community health workers (CHWs), most of whom may not speak English, the multisyllabic foreign word was not appropriate for regular use. In response, a non-profit research group studying CHW referral for mental illness used the term “*gambhir maanasik samasyaa*,” which translates to “severe mental problem”. As a phrase that was insofar not used commonly, it did not carry the level of stigma of other locally used terms that were conceptual equivalents of psychosis. Concurrently, a healthcare organization that was assessing clinician knowledge about mental health used a more technical term “*manobikriti*,” which translates to “abnormality of the mind”. The term is based on roots from Sanskrit, the parent language of Nepali and Hindi, and “*manobikriti*” is often used to denote psychosis in among health workers and educators of health workers in India. However, many Nepali clinicians did not recognize this term to mean psychosis and thus performed poorly on the assessment test. Given that there is no centralized bilingual lexicon, researchers, clinicians, educators, CHWs, and people in the community were all using disparate terms to describe psychosis. Although some variation is expected (e.g., family members or lay community members may use a term that is different from the technical term used by health professionals), this level of variation can lead to poor communication among health professionals and hinders collaborative research, clinical, and training efforts.

Our recommendation is that professionals who engage in clinical translations, particularly in LMICs, first seek out efforts already underway for such work so they can avoid duplication and further fragmentation of efforts [6]. If additional efforts are required to ensure appropriate translations, we suggest coordination with all the stakeholders, particularly ministries of health, to develop a commonly acceptable bilingual lexicon. In most LMICs, the ministry of health is the apex coordinating body and it has the opportunity to facilitate such harmonization. After the 2015 earthquake in Nepal, the WHO and the Nepali government regularly met with all mental health-focused groups as part of a coordinated response to the disaster. This allowed efforts to streamline terminology that is non-stigmatizing and readily recognizable by various stakeholders.

Similarly, producing official translations of treatment guidelines is also an opportunity for harmonization. The World Health Organization (WHO) Mental Health Gap Action Program (mhGAP) has developed two mental health protocols for use in LMICs: the intervention guide (mhGAP-IG) and the humanitarian intervention guide (mhGAP-HIG) [7]. There are number of official translations available from WHO and a large number of other translations are in use throughout the world. With the first version of mhGAP-IG, an *mhGAP Adaptation Guide* provided steps for conducting adaptation workshops and recommended use of an adaptation/contextualization questionnaire to document suggested changes to the guide and training materials. The *Guide* recommends reliance on existing literature and expert opinion to translate clinical terms into terminology that will be standard for communication among non-specialist health workers. Harmonization can also be achieved via collaborations facilitated by online networks such as Guidelines International Network (http://www.g-i-n.net/working-groups/adaptation) and Mental Health Innovations Network (http://mhinnovation.net).

Harmonization of terms is crucial for policy makers because diagnostic categories are tied to access to resources such as health insurance, disability payments, educational accommodations, and to prevent discrimination in employment and other socioeconomic rights. If clinicians and policy makers do not use standardized labels, individuals and families may face additional hurdles to access services and legal protections.

### Terminology for communication between health professionals should be distinct from standardized terminology for communication with patients, families, and the lay public

When translating technical terms, forward-translation (e.g., identifying the local, near-equivalent term for “low mood”) and blinded back-translation (e.g., independently translating the local equivalent back to English to make sure it results in the term “low mood”) are critical steps. To achieve this, professionals often work with people who are not bilingual clinicians because the latter may be familiar with the original text and thus be able to guess the back-translation. However, non-clinical professional translators, without the proper understanding of the context, may conduct literal translations or use highly technical terms that have insofar not been used in clinical settings and are unrecognizable or unacceptable and highly stigmatizing to the lay public. As such, terms that may be acceptable for communication among clinicians may not be appropriate or acceptable to the public.

Therefore, before translations are initiated, the vital first step is to identify the goal of the activity (e.g., clinical care, teaching, research, family psychoeducation, or public awareness raising) [2]. Despite attempts to maximize equivalence, it is important to remember that a completely equivalent translation may simply not occur and will often differ based on the purpose of communication. The goal is not a universal “perfect” translation but translation that avoids harm and meets the patient-oriented clinical, training, and research goals of the interaction.

As an example, when the organization Transcultural Psychiatric Organization (TPO)-Nepal was conducting translation of psychiatric terms in mhGAP and related materials, there was a need to identify terms that could be used with the general public. Terms such as “depression” and “PTSD” can be taught to health workers, but they were not helpful when psychoeducation was needed for community members and patients. Therefore, ethnographic research on conceptualizations of the self, emotions, and social relations (a field of study referred to as *ethnopsychology*) was used to identify appropriate terminology for the purpose of communicating mental health terminology with the lay public. For example, “heart-mind problems” (*manko samasyaa*) and “heart-mind-social problems” (*manosamaajik samasyaa*) were acceptable and could communicate aspect of generalized psychological distress that could be a non-stigmatizing entry point into discussions of potential clinical conditions [3]. When identifying clinically appropriate terms for PTSD, many organizations initially used “mental shock” (*maanasik aaghaat*), however through use of ethnographic research and rapid assessment techniques common in cross-cultural psychology, it was revealed that this term was seen at the intersection of anger (*ris*) and the stigmatizing term for psychosis (*paagal*). Clinical ethnography revealed that “unforgettable tragedy” (*birsana nasakne durghatana*) or “wounded/scarred heart-mind” (*manko ghaau*) were acceptable terms [4]. As such, “PTSD” may be the appropriate term for communication among clinicians, while other ethnopsychologically appropriate terms were better suited for communication with patients and the public.

Our recommendation is to include non-clinicians, including patients and advocacy groups, in the process and ensure that the translation is comprehensible, non-stigmatizing, and otherwise acceptable to them. Another recommendation is to translate not just the actual term but also provide an explanation of the term so that if you are using a technical term, the patient will still understand what it means. TPO Nepal has developed a standardized glossary of terms and definitions that is used for both research and clinical translations (see: https://goo.gl/fiEZXG). This assures organizational continuity across trainings and intervention programs. While implementing mental health care packages in government health services, developing psychoeducation materials and synergizing that language with training manuals helps to promote continuity across practitioners and between training and care provision, as was done by TPO Nepal [8] and *Possible*, a non-profit organization that delivers mental health services along with general healthcare based out of two district-level public hospitals in rural Nepal [9]. If terms for patients and the public are standardized in this way, it also allows for concerted efforts on awareness raising, increasing mental health literacy, reducing stigma, and promoting treatment engagement and adherence.

### Symptom assessment must be linked to functional assessment

Even when health professionals have agreed upon common terminology for assessing symptoms and communicating about mental health with patients, families, and communities, it is important that the terms do not become conflated with disease and disorder without also evaluating functional impairment. As an illustration, when describing the specific symptom of hallucinations as part of a training module for psychosis [9], we received feedback from Nepali generalist clinicians practicing in Nepal that many traditional healers routinely report hearing voices from ancestors, spirits, and other supernatural beings and the clinicians wondered if that meant the healers had psychosis. The healers are not distressed by the symptom and do not have any other signs and symptoms of mental illness. In order to avoid pathologizing this integral part of the local healing tradition, we edited the training module. We elaborated on the “functional impairment” criteria, specifically describing the experience of traditional healers as an example of hallucinations as non-pathological experience. With this edit, the Nepali clinicians felt that “hallucination” would now accurately cover the concept of problematic experiences while leaving out the normative experience of hearing voices in that cultural context. This also allowed us to describe two inter-related concepts: (1) a specific symptom that is largely functional does not imply pathology; and (2) having one symptom does not imply a full illness. This example illustrates that appropriate translation is not simply about identifying the correct terms in the local language, but also about understanding and addressing the contextual environment of the patients.

Ultimately, in clinical settings, the goal of diagnosis is ensuring that normative experiences and behaviors are not pathologized and that distressing symptoms are identified and matched with appropriate treatment recommendations. Therefore, strategies that approach equivalence for symptom assessment and making diagnoses should ensure that these clinical goals are met by assessing functional impairment. Our recommendation to achieve equivalence is to utilize validated tools whenever available. Tools such as the World Health Organization disability assessment scale 12-item short version (WHODAS), the Euroqol-5 dimensions 5 question tool (EQ-5D), and locally developed function tools can provide scores to assess functional impairment and change over time. Clinical frameworks such as the Global Assessment of Functioning (GAF) can also provide a benchmark for impairment and the type and course of treatment.

### Establish a culture of evaluating communication and terminology for continuous improvement

Both lay and professional mental health terminologies continuously change. Standardized language for professional training and communication may need to change as diagnostic and treatment guidelines evolve. Increasing research and clinical care may lead to new diagnostic categories and frameworks, as exemplified by the Chinese Classification of Mental Disorders. Public terminology and public interpretations of mental health terminology are also dynamic. Through a combination of Bollywood memes and increasing acceptance about discussing mental health, the English term “tension” is commonly used to describe stress and distress in Nepal. Therefore, it is crucial to evaluate the terminology used in professional communication and documentation, and the idioms used to describe mental health when engaging with patients and families. Improved professional communication practices may lead to more accurate documentation, increased referrals, and better implementation of policy. Similarly, improvements in professional-to-patient communication can enhance patient’s understanding of the illness, and contribute to treatment engagement and adherence.

Therefore, we recommend documenting communication and evaluating this alongside other markers of quality of care and patient outcomes. For example, the enhancing assessment of common therapeutic factors (ENACT) tool, which was designed and validated in Nepal, includes numerous items evaluating the terminology used in standardized role play conducted at the end of trainings and during supervision sessions [10]. This was used throughout numerous districts to evaluate non-specialist health workers who received mental health training after the 2015 earthquakes. In tandem, the same terms were used by the non-profits *Shared Minds* and *Possible* in developing training materials and clinical protocols for primary care providers in the earthquake affected regions (http://www.sharedminds.org/resources/) [9]. This type of approach enables trainers and supervisors to have not only a common lexicon but also a concrete metric about the application of language in clinical competence.

## Conclusions

Medical education, clinical training, and research practices around the world are increasingly influenced by clinical guidelines, textbooks, ethical frameworks, and research protocols developed in English in HICs. We provide guidance on common challenges in cross-cultural, cross-linguistic settings and suggest that they can be addressed by harmonizing diagnostic and symptom terminology among clinicians, educators, and policy makers; developing distinct terminology to serve the purpose of professionals’ communication with each other versus communication between professionals and patients; linking symptom assessment and functional assessment; and establishing a culture of evaluating communication for continuous improvement. Utilization of these practical tips can assist clinicians, researchers, and educators to participate in culturally-appropriate and humble, cross-linguistic clinical, research, and training efforts in mental health.

## Abbreviations

LMICs: low- and middle-income countries
HIC: high-income country
WHO: World Health Organization
MhGAP: Mental Health Gap Action Program
PHQ-9: patient health questionnaire 9 items
TPO: Transcultural Psychiatric Organization
PTSD: post traumatic stress disorder
CHWs: community health workers
ENACT: enhancing assessment of common therapeutic
